# Association of mastication and factors affecting masticatory function with obesity in adults: a systematic review

**DOI:** 10.1186/s12903-018-0525-3

**Published:** 2018-05-04

**Authors:** Akio Tada, Hiroko Miura

**Affiliations:** 1 Department of Health Science, Hyogo University, 2301 Shinzaike Hiraoka-cho, Kakogawa, Hyogo 675-0195 Japan; 2 Department of International Health and Collaboration, National Institute of Public Health, 2-3-6, Minami, Wako, Saitama, 351-0197 Japan

**Keywords:** Mastication, Obesity, Overweight, Adults

## Abstract

**Background:**

A substantial number of adults suffer from obesity, that is caused by the risk factor, masticatory dysfunction. The association between mastication and obesity, however, is inconclusive. This systematic review aims to provide literature regarding the association between mastication and factors affecting masticatory function, and obesity in adults.

**Methods:**

Four electronic databases (PubMed, EMBASE, Cochrane Library, and Web of Science) were used to search for publications that met the following criteria: published between 2007 and 2016, written in English, and assessed the associations between mastication and obesity among the population aged ≥18 years. The included publications were analyzed based on the study design, main conclusions, and strength of evidence identified by the two authors who screened all the abstracts and full-text articles and, abstracted data, and performed quality assessments by using a critical appraisal tool, the Critical Appraisal Skills Programme Cohort Studies Checklists.

**Results:**

A total of 18 articles (16 cross-sectional, 1 cohort studies, and 1 randomized controlled trial [RCT]) met our inclusion criteria and were evaluated. Poorer mastication was associated with obesity in 12 out of 16 cross-sectional studies. One cohort study showed that the obesity group displayed higher tooth loss than the normal weight group. One RCT demonstrated that gum-chewing intervention for 8 weeks significantly decreased waist circumference.

**Conclusions:**

Most studies revealed a positive association between mastication and obesity among adults. Nonetheless, most of them are cross-sectional studies, which are insufficient to demonstrate a causal relation. Further advancement requires RCT, especially an intervention of improvement of mastication and obesity needed to confirm this association.

**Electronic supplementary material:**

The online version of this article (10.1186/s12903-018-0525-3) contains supplementary material, which is available to authorized users.

## Background

Globally, the prevalence of obesity has increased and is expected to reach around 20% by 2025 if trends in the mean body mass index (BMI), which characterizes its population distribution continue [1]. Obesity causes various health issues. In obese individuals, there is an increased risk of developing type 2 diabetes [2], dyslipidemia [2], hypertension [2], cardiovascular disease [3], fatty liver disease [4], certain types of cancer [5], dementia [6], obstructive sleep apnea [7] and so on. Increases in being overweight and obese reduced life expectancy by 5–13 years [8], increases health care expenditures by 50–200% [9], and dramatically alters quality of life [10]. Increased obesity is a big concern for a person’s medical care and health.

Obesity and being overweight are caused by an energy imbalance between calories consumed and expended [11]. An increased intake of high calorie foods and decreased physical activity due to the increasingly sedentary occupation, and development of transportation are major factors causing energy imbalance. Furthermore, recent obesity has been associated with various factors such as sleeping and smoking [12].

In the last couple of decades, the association between obesity and masticatory function has been noticed, because masticatory function affects nutritional intake [13, 14]. Masticatory function means the objective capacity of a person to tear solid food into pieces or the subjective response of a person to questions concerning chewing food [15]. Objective masticatory function is defined as masticatory performance, which assesses the particle size distribution of food when chewed for a given number of strokes [16] and therefore its impact on obesity is of great concern. However, due to difficulty and variety of objective measurements for mastication, little information is available on the association between masticatory function and obesity. Objective masticatory function has been strongly associated with factors such as the number of remaining teeth, number of missing teeth, and use of prostheses [17–23]. These factors affecting masticatory function have been studied on the association with obesity. Taking this situation into consideration, to review studies for associations both between objective masticatory function and obesity and between factors affecting masticatory function (FAM) and obesity is considered to provide more extensive assessment for the impact of mastication on obesity.

No systematic review article has been published that conducts an overview of a wide range of literature describing this association. Further progression of studies in this field requires an overview of the literature.

In this review article, we provide a literature overview on the association of obesity with objective masticatory function and FAM.

## Methods

### Literature search

The investigation was conducted by searching four electronic databases (PubMed, EMBASE, Cochrane Library, and Web of Science), using the following terms: (“chewing” OR “number of teeth” AND “obesity”), (“mastication” OR “number of teeth” AND “obesity”) and (“masticatory performance” AND “obesity”). Two authors (AT and HM) independently assessed each retrieved document for eligibility by examining the titles and abstracts, based on the inclusion and exclusion criteria shown in Table 1. Since subjective masticatory function includes adaptive and psychological factors, the range of mastication was limited to objective masticatory function. We defined the FAM as dentition and salivary flow rate, which follows the criteria written in a review article previously reported [15]. Epidemiological studies that investigated the association between mastication and obesity in adults published between 2007 and 2016 were included. Additional inclusion criteria were (1) studies in adult subjects (age ≥ 18 years); (2) studies written in English. The papers were excluded from the systematic review if (1) studies that were conducted in subjects who received oral and maxillofacial surgery or radio therapy; (2) studies that were conducted in people with systemic illness; (3) descriptive studies, review, or studies with no analyses investigating the association between mastication and obesity.

**Table 1:** Inclusion and exclusion criteria used in this review

	Inclusion criteria	Exclusion criteria
Sample	Subjects aged 18 years or older,	Subjects who received oral and maxillofacial surgery or, radiotherapy Subjects who have systemic illness
Language	Written in English	Not written in English
Analysis	Any association between mastication and obesity	Descriptive studies, review, or studies with no analyses investigating the association between mastication and obesity

### Quality assessments

The Critical Appraisal Skills Programme Cohort Studies Checklists [24] was used for quality assessment. The checklist for cohort studies was modified for application to cross-sectional studies (e.g. Question 2, “Was the cohort recruited in an acceptable way?” was modified to “Was the sample recruited in an acceptable way?”, and questions regarding follow-up of participants were excluded). For each study, the strength and weaknesses were calculated based on the relevant checklist items and a grade of “low”, “moderate,” or “high” was assigned with the two authors’ (AT and HM) approval.

### Data extraction

Two authors extracted information independently, and disagreements were resolved by consensus. The following data were extracted from each eligible study: first author, year of publication, country where the study conducted, study type, study period, number of cases, confounding factors, and both adjusted odds rate (95% CI).

## Results

### Literature searches and study characteristics

**Fig. 1:**
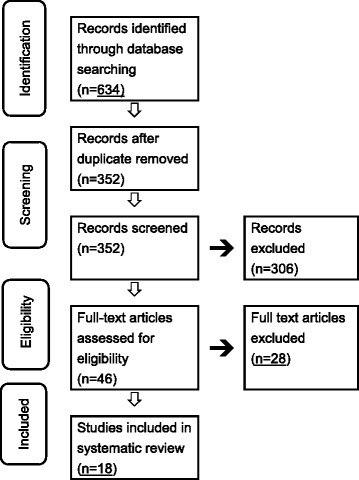
Flow diagram of literature search

Our initial search identified a total of 634 publications, as shown in flow chart (fig. 1). After removal of duplication and title and abstract screening, 46 articles were selected for full-text screening. The full-text articles of the 46 potentially relevant references were reviewed, 28 of which did not fit the inclusion criteria and were consequently excluded. Finally, 18 publications (16 cross-sectional studies, 1 cohort studies, and 1 randomized controlled trial [RCT]) were selected as the “key articles”, that would subsequently be scrutinized for the study design.

Of the 18 included studies, except the RCT, 7 studies evaluated masticatory function (masticatory performance) [25–31], 10 factors affecting masticatory function (the number of teeth, number of missing teeth, use of prostheses, chewing-gum-stimulated saliva flow) [32–41]. In this article, we systematically review the published findings on the associations of obesity with masticatory function and FAM.

With regard to obese and overweight criteria, 16 studies measured BMI, 8 waist circumference (WC) and 5 waist-to-hip ratio (WHR).

### Quality of studies

Additional file 1: Table S1 presents the results of the critical appraisal assessment. Recurrent strengths of the evidence included the following: (1) addressing a clearly focused issue (*n* = 18; 100%); (2) recruiting subjects in an acceptable manner (cross-sectional *n* = 14 [87.5%], cohort *n* = 1 [100%], and RCT n = 1; [100%]); (3) measuring exposure to minimize bias (cross-sectional *n* = 15 [93.8%], cohort *n* = 1 [100%], and RCT *n* = 1; [100%]), and (4) measuring outcome to minimize bias (*n* = 18; 100%).

The most common issue for critical appraisal that was observed across studies was not adjusting all potential confounding factors. Only three studies adequately adjusted their analyses for all the potential confounders, such as the socio-demographic and, socioeconomic factors, and health habits including physical activity for obesity [32, 33, 41].

Overall, the quality of the included articles were “High” (4 studies: 2 cross-sectional, 1 cohort, and 1 RCT) to “Moderate” (11 cross-sectional) with only 3 studies judged as being “Low” (2 cross-sectional studies for masticatory function, 1 cross-sectional study for FAM).

### Impact of mastication on obesity

Additional file 1: Table S2 describes the findings regarding the association between mastication and obesity from the studies.

#### Cross-sectional studies for masticatory function

Seven cross-sectional studies investigated the association between masticatory function and obesity [25–31]. All studies used BMI as an obesity indicator. Three studies demonstrated that participants with lower masticatory function had significantly higher BMI [25, 27, 30]. One study showed that the obese group exhibited worse masticatory function than other anthropometric groups in men but not in women [28]. However, three showed no association [26, 29, 31].

#### Cross-sectional studies for FAMs

Nine studies analyzed the associations between FAM and obesity [32–40]. The findings from five of these studies showed a significant association between the number of teeth / missing teeth and BMI [32, 36, 37, 39, 40]. One study showed no association between the number of teeth and BMI [33]. Five studies showing higher number of teeth had lower WC/WHP [32, 33, 35, 36, 38]. One study examined the associations between the masticatory ability evaluated using a chewing-gum-stimulated salivary flow rate and anthropometric indices [34].

#### Longitudinal studies for masticatory function and FAM

One cohort study and one RCT showing the relationship between mastication and obesity were found. One prospective cohort study showed that tooth loss was higher among men and women with the third tertile of WHR (Men: IRR = 1.37, 95% CI = 1.04 to 1.80; Women: IRR = 1.53, 95% CI = 1.14 to 2.05) [41]. It is discussed that periodontal disease is involved in the association between tooth loss and obesity. One RCT showed that an intervention of gum-chewing and printed nutrition information for 8 weeks significantly decreased the WC [42]. However, the control group which received only printed nutrition information presented a little decrease in WC. A significant effect might not be obtained only by gum-chewing.

## Discussion

### Quality assessment of the studies

One important finding of this review is the use of objective indicators for mastication and obesity in almost all studies. One study used the self-reported number of missing teeth [36]. Since subjective measurements yield much more optimistic results than practitioner’s measurements do [43], there is a possibility that self-reported number attenuated the accuracy of the data.

Cut-off values of the number of teeth for categorization were differential and likely depend on ethnicity, with ≥20/21 versus < 20/21 for European [32, 40] and ≥ 8/10 versus < 8/10 for Brazilian people [36, 39]. This difference may be due to the consideration of the level of oral health in each country. Since the number of teeth does not have any international standard like BMI (25 for overweight, 30 for obese) [44], its cut-off values in key articles differ widely among studies. Therefore, an international standard to categorize oral health with regard to the number of teeth will be beneficial for oral epidemiological studies.

With regard to masticatory function, five key articles used particle size [26–29, 31], and one chewing mixing method [25]. Other than these methods, masticatory function assessments generally used questionnaires, kinematics, and video recording, as indirect methods, and chewing activity, and maximum bite force, as direct methods. These different methods make comparison of the masticatory function levels among studies difficult.

Cut-off values of BMI used in key articles were ≥ 25 [25, 39], ≥25 and ≥ 30 [40] and ≥ 30 [33, 37], which follow the WHO standards [44]. Studies conducted in the Japanese population used ≥25 as the cutoff value, based on the criteria established by the Japan Society for obesity [45]. In general, a smaller percentage of Japanese have a BMI of ≥30. Careful attention is necessary when comparing the associations between Japanese people and other ethnic population. Cut-off values of WC used were > 102 cm for men- and > 88 cm for women [33, 36], > 94 cm for men and > 80 cm for women [35, 38], and > 88 cm for both [32]. Although WC is influenced by ethnicity, four articles investigated Brazilians. Although detailed information on ethnicity in these studies are not available, double standards in one country may affect the outcome.

The majority of obesity cases are thought to be explained by a combination of excessive food energy intake and lack of physical activity [46]. Furthermore, other possible factors have been identified to contribute to the recent increase of obesity [12]. Although socio-demographic factors were used in 12 of the 17 studies, lifestyle variables, including physical activity were employed in only six studies. Energy intake was adjusted for by only one key article probably due to the need for professional staff during evaluation. Moreover, five key articles did not adjust for confounding factors [26, 28–30, 37]. The lack of adjustment of these factors would possibly lead to overestimation or underestimation of the relationship between mastication and obesity.

### Relationship between mastication and obesity

The association between tooth loss/ edenturism and obesity was reviewed previously [47]. Our study has a strength to review studies concerning association between masticatory function and obesity in addition to that between tooth loss/ edenturism and obesity. Most key articles in our studies evaluating the number of teeth and obesity showed significant association. This result is consistent with observation in Nascimento’s systematic review [47]. However, four out of eight studies failed to observe the association between masticatory function and obesity [26, 28, 29, 31]. These studies investigated participants aged ≤40 yrs. Almost all studies including participants aged ≥50 showed significant associations between masticatory function or factors affecting masticatory function and obesity. The influence of mastication on obesity may not be significant in younger population because they have higher physical activity and basal metabolic than older population do. However, there is one study that failed to find this association in older people [48]. Furthermore, since the significant association disappeared by stringent adjustments in some key articles, the significance of association might not be necessarily great.

One prospective cohort study revealed that obese group had higher tooth loss than normal group does [41], suggesting that obesity causes tooth loss. However, another causal relationship showing that decreased mastication causes obesity should be investigated. Prospective cohort studies that observe and compare changes in the body weight among participants with differential masticatory function are necessary. Although RCT is necessary to reveal causal relationship, only one has been available in this association. More intervention studies should be conducted to verify the impact of mastication on obesity. Especially, intervention studies that examine the body weight changes in participants who are provided prosthetics and experience restoration of mastication will provide beneficial information to determine the causal relationship between obesity and chewing status.

Of the three key articles that measured both BMI and WC, two found higher odds ratio for the associations between the number of teeth and WC than those with BMI [33, 36], but one displayed similar odds ratio for both [32]. The participants of the former were Brazilian and those of the latter were Germans. The difference might be explained by ethnicity, but it needs more studies conducted in differential ethnic. Since abdominal obesity is related to metabolic syndrome [49], further research regarding relationship between mastication and obesity (general and abdominal obesity) is of great concern.

**Fig. 2:**
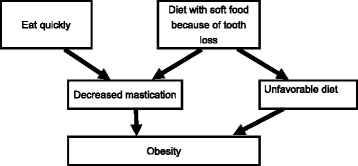
Schema for the association between mastication and obesity

There are two possible reasons which explain the association between mastication and obesity (fig. 2). One is that people with poor masticatory function tend to have smaller consumption of vegetable and fruit, and higher consumption of high energy food than those with adequate mastication [50–53], which causes obesity. Another is that less chewing leads to obesity-causing-phenomena (decrease in diet induced thermogenesis and inactivation of neuronal histamine) [54–56]. Small number of chewing strokes decreases the effect of chewing to prevent obesity. Diet with soft food due to decreased masticatory ability decrease the number of chewing cycle. Other than that, quickly eating, although being a problem of eating behavior, decreases number of chewing cycle. Several cross-sectional studies [57–59] showed that people who tend to eat quickly have increased BMI than those who ate slowly. Yamane et al. demonstrated this association in a longitudinal prospective study [60]. One cohort study showed that the lowest group in the number of chewing cycles during meals had a significantly higher rate of incremental increases in body weight than the highest group [61]. Moreover, this association was reviewed in a systematic review [62]. Robinson argued that a slower eating rate was associated with lower energy intake in comparison to a faster eating rate in a systematic review [63]. These studies support that lower number of chewing cycle is associated with obesity. On the other hand, there were reports showing that chewing rate and eating behavior did not differ between people with high and normal BMI [64, 65]. A study reported that anthropometric status affect energy intake after chewing, which makes association between mastication and obesity complex [66].

Findings of key articles is considered to be responsible for these mechanisms. The former mechanism is assumed to give considerable influence on the studies in this review with subjects of older adults.

### Association among obesity, mastication and periodontal disease

**Fig. 3:**
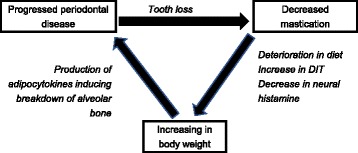
Schema of vicious circle

Periodontal disease is an essential factor for considering the association between mastication and obesity because it is strongly linked to mastication and obesity. Obesity is considered one of the factors that cause incidence and progression of periodontal disease [67]. Proinflammatory adipocytokines such as TNF-α, which is produced, primarily in the abdominal adipose tissue, induces the breakdown of alveolar bones, in turn periodontal tissue degradation [68, 69]. Periodontal disease is one of the major causes of tooth loss because it destroys the tissue supporting the teeth, which deteriorate masticatory function. Most elderly people with decreased mastication are considered to have periodontitis. The key articles observed a significant association between masticatory function and obesity in younger populations in which severe periodontal disease are supposed to be less prevalent, suggesting that mastication influenced obesity independent of the presence of periodontal disease [25, 27]. Given that decreased mastication increases obesity, the following vicious circle is hypothesized in elderly people (Fig. 3). Obesity causes periodontal disease progression, subsequently, in turn, leading to deterioration of mastication caused by tooth loss. Furthermore, decreased mastication progresses obesity. Moreover, it is guessed that the association between periodontitis and obesity is bidirectional [70–72]. Endotoxin from Gram negative bacilli have been reported to be responsible for body weight gain and diabetes [73] which suggests the possibility of induction of obesity by endotoxin of periodontal pathogens. There may be complicated cascades among mastication, periodontal disease and obesity. In a study by Meisel et al., inflammatory materials are discussed to mediate the association between tooth loss and obesity [41]. Analysis of the influence of oral health on obesity will need approaches from both mastication perspective and periodontal perspective.

### Limitation

There are several limitations in this study. First, only a small number of intervention and cohort studies were found. These longitudinal studies, particularly intervention study, are necessary to analyze the causal relationships. Second, less than half studies used the masticatory function as indicator of mastication. Although a low number of natural teeth has been reported to be associated with a poor chewing function [74, 75], it does not necessarily show a close relation with mastication [76]. This can be explained by the fact that chewing efficiency differs depending on the location of the missing teeth among individuals with the same number of teeth. Third, although obesity is strongly related to physical activity and energy intake, more than half of studies did not adjust these factors in calculating the odds ratio for the association between mastication and obesity, or did not even do adjustment any factors. This insufficient adjustment prevents accurate assessment of the association. Finally, many studies were conducted in Japan or Brazil, preventing generalization of the results. Therefore, since mastication is influenced by the dietary culture, integrated evaluation of results from various areas is necessary.

### Future direction

More intervention studies are necessary to elucidate the association between mastication and obesity. In particular, prospective cohort and intervention studies are necessary to analyze if decreased mastication leads to obesity and investigate if provision of prosthesis will work to improve obesity, respectively.

Cross sectional and cohort studies revealing the association between mastication and obesity should be analyzed by adjusting the energy intake and physical activity. Studies regarding the relationship between mastication and dietary/nutrient intake have been published [14, 77–79]. Adding obesity measurement in these investigations would be possible.

Since mastication is the process of crushing and grounding the food using the teeth, it should be evaluated using functional parameters such as masticatory function rather than the number of anatomically present teeth. The measurement of mastication function is desirable to be easy and reliable. Moreover, a unified assessment method should be used to compare these studies. Chewing gum mixing method has made mastication assessment easy and made the evaluation of large-scale participants in a short period of time [80, 81] and its reliability and validity of the color scale to evaluate the chewing ability have been previously reported [82]. It may be used more frequently to evaluate the mastication in large-scale studies to elucidate the impact of mastication on obesity in the future.

In elderly people, progression of periodontal disease facilitates decreased masticatory function, which in turn, increases obesity. Moreover, periodontopathogens have been suggested to induce obesity. Regression models and meditation analysis should be used to elucidate the association among mastication, periodontal disease and obesity.

## Conclusion

Most cross-sectional studies show that poor mastication was associated with obesity. Two prospective cohort studies and RCT demonstrated that poorer mastication is one of the risk factor of obesity. These studies suggest that mastication may have close relationship with obesity. Further research, especially involving intervention studies are required to improve our current understanding of the relationship of mastication and obesity.

## Additional file

Additional file 1: **Table S1**. The results of the critical appraisal assessment. **Table S2**. Summary of studies on the relationship between mastication and AMF and obesity. (DOCX 31 kb)
