# Supplementary material for: Association of mastication and factors affecting masticatory function with obesity in adults: a systematic review
Source: BMC Oral Health. 2018 May 4;18:76. doi: 10.1186/s12903-018-0525-3 (PMC5935987; doi:10.1186/s12903-018-0525-3)
Supplement: Supplementary file 1 — Table S1. The results of the critical appraisal assessment. Table S2. Summary of studies on the relationship between mastication and AMF and obesity. (DOCX 31 kb) [file 12903_2018_525_MOESM1_ESM.docx]

Table S1. The results of the critical appraisal assessment

1) Cross-sectional study for masticatory function

|  | 1 | 2 | 3 | 4 | 5a | 5b | 6 | 7 | 8 | Quality assessment |
| --- | --- | --- | --- | --- | --- | --- | --- | --- | --- | --- |
| Katagiri *et al.* (2011) | ✓ | X | ✓ | ✓ | X | X | ✓ | X | ✓ | Low |
| Flores-Orozco *et al.* (2016) | ✓ | ✓ | ✓ | ✓ | X | X | ✓ | ✓ | ✓ | Moderate |
| Sánchez-Ayala *et al.* (2013) | ✓ | X | ✓ | ✓ | X | X | ✓ | X | ✓ | Low |
| Isabel *et al.* (2015) | ✓ | ✓ | ✓ | ✓ | X | X | ✓ | ✓ | ✓ | Moderate |
| Frecka *et al.* (2008) | ✓ | ✓ | ✓ | ✓ | X | X | ✓ | ✓ | ✓ | Moderate |
| Zijlstra *et al.* (2011) | ✓ | ✓ | ✓ | ✓ | X | X | ✓ | ✓ | ✓ | Moderate |
| Carvalho *et al*. (2016) | ✓ | ✓ | ✓ | ✓ | X | X | ✓ | ✓ | ✓ | Moderate |

2) Cross-sectional study for factors affecting masticatory function

|  | 1 | 2 | 3 | 4 | 5a | 5b | 6 | 7 | 8 | Quality assessment |
| --- | --- | --- | --- | --- | --- | --- | --- | --- | --- | --- |
| Peruchi *et al.* (2016) | ✓ | ✓ | ✓ | ✓ | X | X | ✓ | ✓ | ✓ | Moderate |
| Östberg *et al.* (2012) | ✓ | ✓ | ✓ | ✓ | ✓ | ✓ | ✓ | ✓ | ✓ | High |
| Singh *et al.* (2016) | ✓ | ✓ | ✓ | ✓ | ✓ | ✓ | ✓ | ✓ | ✓ | High |
| Sheiham *et al.* (2002) | ✓ | ✓ | ✓ | ✓ | X | X | ✓ | ✓ | ✓ | Moderate |
| Prpić *et al.* (2012) | ✓ | ✓ | ✓ | X | X | X | ✓ | ✓ | ✓ | Moderate |
| De Marchi *et al.* (2012) | ✓ | ✓ | ✓ | ✓ | X | X | ✓ | ✓ | ✓ | Moderate |
| Hilgert *et al.* (2009) | ✓ | ✓ | ✓ | ✓ | X | X | ✓ | ✓ | ✓ | Moderate |
| Bernardo Cde *et al.* (2012) | ✓ | ✓ | X | ✓ | X | X | ✓ | ✓ | ✓ | Low |
| Maruyama *et al.* (2015) | ✓ | ✓ | ✓ | ✓ | X | X | ✓ | ✓ | ✓ | Moderate |

✓, satisfied; X, not satisfied.

1 Did the study address a clearly focused issue?

2 Were the subjects recruited in an acceptable way?

3 Was the exposure accurately measured to minimise bias?

4 Was the outcome accurately measured to minimise bias?

5 (a) Have the authors identified all important confounding factors?

5 (b) Have they taken account of the confounding factors in the design and/or analysis?

6. Do you believe the results?

7. Can the results be applied to the local population?

8. Do the results of this study fit with other available evidence?

3) Cohort

|  | 1 | 2 | 3 | 4 | 5a | 5b | 6a | 6b | 7 | 8 | 9 | Quality assessment |
| --- | --- | --- | --- | --- | --- | --- | --- | --- | --- | --- | --- | --- |
| Meisel *et al.* (2014) | ✓ | ✓ | ✓ | ✓ | ✓ | ✓ | ✓ | ✓ | ✓ | ✓ | ✓ | High |

✓, satisfied; X, not satisfied.

1-5b: The same as cross-sectional study

6a. Was the follow up of subjects complete enough?

6b. Was the follow up of subjects long enough?

7. Do you believe the results?

8. Can the results be applied to the local population?

9. Do the results of this study fit with other available evidence?

4) RCT

|  | 1 | 2 | 3 | 4 | 5 | 6 | 7 | 8 | 9 | Quality assessment |
| --- | --- | --- | --- | --- | --- | --- | --- | --- | --- | --- |
| Shikany *et al.*, (2012) | ✓ | ✓ | ✓ | ✓ | ✓ | ✓ | ✓ | ✓ | ✓ | High |

✓, satisfied; X, not satisfied.

1. Did the trial address a clearly focused issue?

2. Was the assignment of patients to treatments randomised?

3. Were all of the patients who entered the trial properly accounted for at its conclusion?

4. Were patients, health workers and study personnel ‘blind’ to the treatment?

5. Were the groups similar at the start of the trial?

6. Aside from the experimental intervention, were the groups treated equally?

7. Can the results be applied in your context?

8. Were all clinically important outcomes considered?

9. Are the benefits worth the harms and costs?

Table S2. Summary of studies on the relationship between mastication and AMF and obesity

1) Cross-sectional study for masticatory function

| Reference | Study sample | Measure for mastication | Measure for outcome | Control of confounding factors^a^ | | Key results | |  |
| --- | --- | --- | --- | --- | --- | --- | --- | --- |
| Katagiri *et al*. (2011) | 173 individuals (75 obese (34 males and 41 females) and 98 normal weight (63 males and 35 females), 25-40 yrs, Japan) | Chewing gum mixing method | Body mass index (BMI), | 1 | | Significant correlation between obesity and reduced masticatory function | |  |
| Flores-Orozco *et al.* (2016) | 100 Individuals (mean age 21.9 yr, Mexico) | Particle size measurement | BMI |  | | No association-masticatory performance and obesity | |  |
| Sánchez-Ayala *et al.* (2013) | 110 dentate and partially edentulous participants (mean age 39.7 yrs, Brazil) | Particle size measurement | BMI | 1 | | Positive association-lower masticatory efficiency and BMI (OR = 4.792, 95% CI = 1.419- 16.183) | |  |
| Isabel *et al.* (2015) | 160 individuals  (18-40 yrs, Brazil) | Particle size measurement | BMI |  | | Male: obese group showed the worst masticatory performance.  Female: No association-  Masticatory performance and obesity | |  |
| Frecka *et al.* (2008) | 24 individuals (12 lean (BMI=22.2+/-0.3) and 12 obese (BMI=34.3+/-0.6), mean age 25.2 yr, USA) | Particle size measurement | BMI |  | | No association | |  |
| Zijlstra *et al.* (2011) | 54 individuals (27 normal weight and 27 overweight, 18-55 yrs, Netherland) | Bite size | BMI |  | | Mean bite size for spiced rice was significantly (P = 0.03) larger in overweight/obese (10.3 (SD 3.2) g) v.s. normal-weight subjects (8.7 (SD 2.1) g). | |  |
| Carvalho *et al*, (2016) | 171 individuals (46 males and 125 females, 18-33 yrs, Brazil) | Particle size measurement | BMI and  Waist circumference (WC) | | 1 | | No association-masticatory performance and BMI and WC | |

2) Cross-sectional study for factors affecting masticatory function

| Reference | Study sample | Measure for mastication | Measure for outcome | Control of confounding factors^a^ | Key results |
| --- | --- | --- | --- | --- | --- |
| Peruchi *et al.* (2016) | 489 individuals (60 yrs and over, Brazil) | Number of teeth | WC and  Waist-hip ratio (WHR) | 1,2,3 | Positive association-WHR and having fewer natural teeth (OR = 2.61; 95%CI = 1.17-5.80), being edentulous and wearing both upper and lower complete dentures (OR = 2.34; 95%CI = 1.11-4.93), and being edentulous wearing only the upper complete denture (OR = 2.64; 95%CI = 1.01-6.95). |
| Östberg *et al.* (2012) | 999 individuals (38-78 yrs, Sweden) | Number of teeth and number of restored teeth | BMI, WHR and WC | 1,2,3,4 | Positive associations- a small number of teeth (<20) and obesity: BMI (OR 1.95; 95% CI 1.40-2.73), WHR (1.67; 1.28-2.19) and waist circumference (1.94; 1.47-2.55) |
| Singh *et al.* (2016) | 1,704 individuals (60 yrs and over, Brazil) | Number of teeth | BMI and WC | 1,2,3,4 | No association for BMI  Positive association for WC-Edentate (OR1.5; 95%CI 1.0-2.4), |
| Sheiham *et al.* (2002) | 629 individuals (65 yrs and over, UK) | Number of teeth | BMI | 1,2, | Positive association-  11-20 (OR 3.3; 95%CI 1.29-8.22)  1-10 (3.1; 1.08-8.84) |
| Prpić *et al.* (2012) | 320 non-smoking individuals (31-60 yrs, Croatia) | Number of missing teeth | BMI |  | Multivariate linear analysis showed that BMI was most dependent upon the number of missing teeth (88.6%) |
| De Marchi *et al.* (2012) | 471 individuals (60-89 yrs, Brazil) | Number of teeth | WC and WHR | 1,2,3 | Participants with more than 8 teeth were less likely to have central obesity, as measured by WHR [OR = 0.49 (0.32 to 0.87)], while participants with only 1-8 natural teeth were more likely to have central obesity when evaluated by WC [OR = 3.28 (CI 1.43 to 7.52)]. |
| Hilgert *et al.* (2009) | 872 individuals (60 yrs and over, Brazil) | Number of teeth and use of prostheses | BMI | 1,2,3 | Multivariate logistic regression revealed that edentulous persons wearing only upper dentures (OR = 2.34, 95% CI 1.18-4.27) and dentate participants with one to eight teeth wearing 0-to-1 prosthesis (OR = 2.96, 95% CI 1.68-5.19) were more likely to be obese. |
| Bernardo Cde *et al.* (2012) | 1,720 individuals (20-59 yrs, Brazil) | Self-reported number of teeth | BMI and WC | 1,2 | Positive association- < 10 teeth in at least one arch and BMI and WC (depended on age group) |
| Maruyama *et al.* (2015) | 921 individuals (30-79 yrs, Japan) | Chewing-gum-stimulated salivary flow rate | BMI, WC, WHR and  Skinfold thickness | 1,3,4 | Negative association-higher saliva flow and BMI (OR =0.59, 95%CI = 10.37-0.95), and WC (OR = 0.65; 95%CI = 0.43-0.98), and WHR (OR=0.54, 95%CI=0.35-0.83), and Skinfold thickness OR=0.61, 95%CI=0.39-0.96) |

3) Cohort study

| Reference | Study sample | Measure for mastication | Measure for outcome | Control of confounding factors^a^ | Key results |
| --- | --- | --- | --- | --- | --- |
| Meisel *et al* (2014) | 2,714 individuals (20-80 yrs at base line, Germany) | Tooth loss | BMI and WHR | 1,2,3,4 | Adjusted for covariates, the IRR of tooth loss associated with the third tertile of waist-to-hip ratio was 1.37 (95% CI: 1.04, 1.80) and 1.53 (95% CI: 1.14, 2.05) in men and women, respectively. |

4) RCT

| Reference | Study sample | Measure for mastication | Measure for outcome | Control of confounding factors^a^ | Key results |
| --- | --- | --- | --- | --- | --- |
| Shikany *et al*., (2012) | 201 overweight and obese adults | Gum-chewing protocol in the intervention group | Change in BMI and WC | * | No changes in weight or BMI in either group between baseline and the end of the intervention at 8 weeks.  Significant decrease in waist circumference d in the intervention group between baseline and 8 weeks (mean ± SD change = -1.4 ± 5.3 cm; P = 0.0128); No significant difference in change in control group. |

^a^ The following variables were controlled for in the analyses or with separate results: 1, demographic factors; 2, socio-economic factors; 3, smoking/alcohol; 4, exercise/physical activity

* Not applicable to adjustment with confounding factors
